# Nanotoxicology and Nanosafety: Safety-by-Design and Testing at a Glance

**DOI:** 10.3390/ijerph17134657

**Published:** 2020-06-28

**Authors:** Aleksandra Zielińska, Beatriz Costa, Maria V. Ferreira, Diogo Miguéis, Jéssica M. S. Louros, Alessandra Durazzo, Massimo Lucarini, Piotr Eder, Marco V. Chaud, Margreet Morsink, Niels Willemen, Patrícia Severino, Antonello Santini, Eliana B. Souto

**Affiliations:** 1 Department of Pharmaceutical Technology, Faculty of Pharmacy, University of Coimbra, Pólo das Ciências da Saúde, Azinhaga de Santa Comba, 3000-548 Coimbra, Portugal; zielinska-aleksandra@wp.pl (A.Z.); beafecosta@gmail.com (B.C.); mariavferreira00@gmail.com (M.V.F.); diogodmigueis@gmail.com (D.M.); jessicamslouros@gmail.com (J.M.S.L.); 2 Institute of Human Genetics, Polish Academy of Sciences, Strzeszyńska 32, 60-479 Poznań, Poland; 3 CREA-Research Centre for Food and Nutrition, Via Ardeatina 546, 00178 Rome, Italy; alessandra.durazzo@crea.gov.it (A.D.); massimo.lucarini@crea.gov.it (M.L.); 4 Department of Gastroenterology, Dietetics and Internal Diseases, Poznan University of Medical Sciences, Przybyszewskiego 49, 60-355 Poznań, Poland; piotr.eder@op.pl; 5 Laboratory of Biomaterials and Nanotechnology, University of Sorocaba—UNISO, Sorocaba 18023-000, Brazil; marco.chaud@prof.uniso.br; 6 Center for Biomedical Engineering, Department of Medicine, Brigham and Women& Hospital, Harvard Medical School, 65 Landsdowne Street, Cambridge, MA 02139, USA; m.a.j.morsink@student.utwente.nl (M.M.); n.g.a.willemen@student.utwente.nl (N.W.); pattypharma@gmail.com (P.S.); 7 Translational Liver Research, Department of Medical Cell BioPhysics, Technical Medical Centre, Faculty of Science and Technology, University of Twente, 7522 NB Enschede, The Netherlands; 8 Department of Developmental BioEngineering, Faculty of Science and Technology, Technical Medical Centre, University of Twente, 7522 NB Enschede, The Netherlands; 9 Nanomedicine and Nanotechnology Laboratory (LNMed), Institute of Technology and Research (ITP), University of Tiradentes (Unit), Av. Murilo Dantas, 300, Aracaju 49010-390, Brazil; 10 Tiradentes Institute, 150 Mt Vernon St, Dorchester, MA 02125, USA; 11 Department of Pharmacy, University of Napoli Federico II, 80131 Napoli, Italy; 12 CEB—Centre of Biological Engineering, University of Minho, Campus de Gualtar, 4710-057 Braga, Portugal

**Keywords:** nanotoxicology, nanosafety, nanomaterials, risk assessment, toxicity tests, nanoparticles, human health, Safety-by-Design, biological systems

## Abstract

This review offers a systematic discussion about nanotoxicology and nanosafety associated with nanomaterials during manufacture and further biomedical applications. A detailed introduction on nanomaterials and their most frequently uses, followed by the critical risk aspects related to regulatory uses and commercialization, is provided. Moreover, the impact of nanotoxicology in research over the last decades is discussed, together with the currently available toxicological methods in cell cultures (in vitro) and in living organisms (in vivo). A special focus is given to inorganic nanoparticles such as titanium dioxide nanoparticles (TiO_2_NPs) and silver nanoparticles (AgNPs). In vitro and in vivo case studies for the selected nanoparticles are discussed. The final part of this work describes the significance of nano-security for both risk assessment and environmental nanosafety. “Safety-by-Design” is defined as a starting point consisting on the implementation of the principles of drug discovery and development. The concept “Safety-by-Design” appears to be a way to “ensure safety”, but the superficiality and the lack of articulation with which it is treated still raises many doubts. Although the approach of “Safety-by-Design” to the principles of drug development has helped in the assessment of the toxicity of nanomaterials, a combination of scientific efforts is constantly urgent to ensure the consistency of methods and processes. This will ensure that the quality of nanomaterials is controlled and their safe development is promoted. Safety issues are considered strategies for discovering novel toxicological-related mechanisms still needed to be promoted.

## 1. Introduction

Nanomaterials (NMs) are natural or manufactured materials based on nanosized particles in a disaggregated state or in the form of an aggregate/agglomerate [1]. The number–size distribution of 50% or more of the particles which have one or more external dimensions in the size range comprised between 1 and 100 nanometers [2,3]. Due to the small size of the particles and changes in their inner structure, NMs can have different properties that stem from a higher surface area to volume ratio [4]. Therefore, the physicochemical properties of NMs may differ from the properties of granular substances or larger particles [5]. 

**Figure 1:**
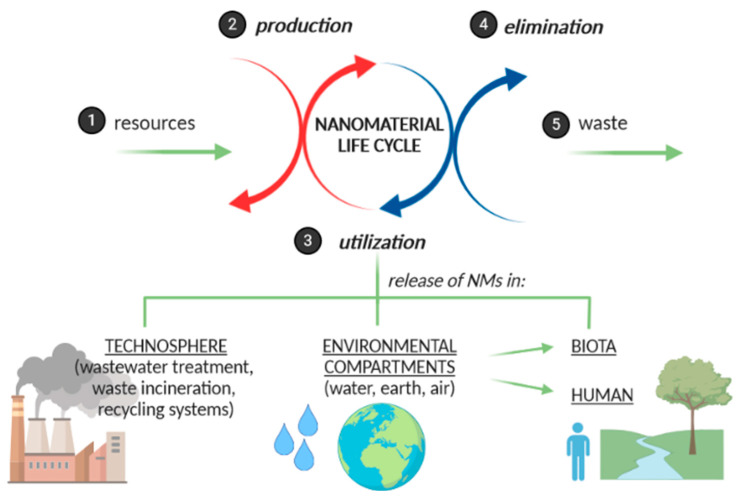
The impact of nanomaterial life cycle on the environment.

There is a huge demand for a definition of NMs [6,7]. On the other hand, the life cycle assessment of NMs has been recognized as a significant tool for regularly evaluating the potential environmental impacts of manufactured nanomaterial during their complete life cycles [8], and it is schematically presented in Figure 1.

**Figure 2:**
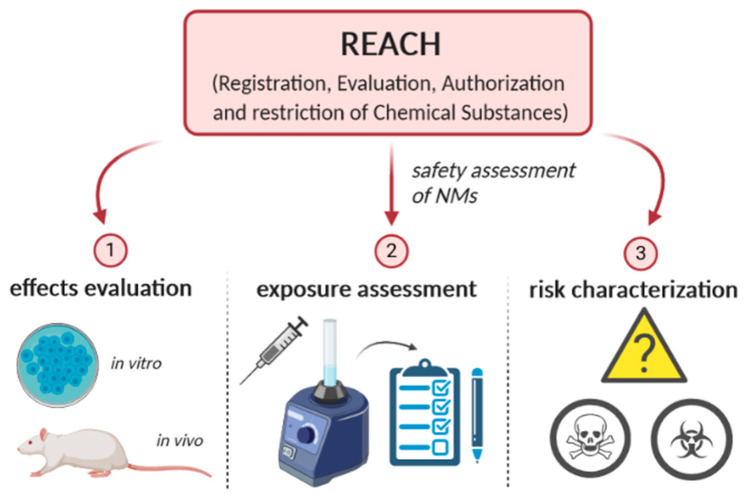
The risk assessment methodology for nanomaterials (NMs) based on Registration, Evaluation, Authorization and restriction of Chemical Substances (REACH) requirements.

For a long time, it has been assumed that NMs have a similar toxicity to materials of larger size [9]. However, studies have shown that nanosized materials exhibit different physicochemical properties from those of the source material (thereby changing their reactivity in biological systems). It calls into the question whether applying conventional methodologies to evaluate the adverse effects of NMs is still valid [10]. According to “REACH” (Registration, Evaluation, Authorization and restriction of Chemical Substances), the safety assessment of NMs should follow the risk assessment methodology adopted for conventional chemicals, which is based on the following requirements [11]: (1) effects evaluation, (2) exposure assessment, and (3) risk characterization (Figure 2). Step 1 includes the evaluation of effects. The risk quotient is considered acceptable (higher than 1) when the estimated exposure value is lower than the concentration of the agent, in which no adverse effect was observed in the experimental study carried out to evaluate the point under study—for example, inhalation toxicity or genotoxicity. If it is necessary to carry out in vitro and/or in vivo experiments in order to assess the effects, other procedures to characterize the NMs under study may also be recommended. This includes collecting information on the most relevant physicochemical parameters that may influence the toxicity, i.e., size distribution, aggregation/agglomeration status, shape, surface area, reactivity, water solubility, surface properties, and long-term stability [11,12]. Step 2 includes the identification of all potential sources of exposure [13]. Thus, it is important to understand the full manufacturing process and the most likely routes of exposure. This is also relevant for choosing the appropriate testing strategy and for making recommendations on risk prevention measures (Step 3) [14].

The NanoRoadMap Project, funded by the European Commission, has develop guidelines for the nanotechnological development in three major areas, namely Energy, Materials and Health, and Medical Systems. The project created new possible applications of NMs, as described in this fragment: *“with the development of current and emerging technology to manipulate matter on a nanoscale and atomic scale, we have reached a point where any viable molecular structure to include a large number of nuclides can be designed and built at will”* [6]. Therefore, NMs are nowadays applied in multiple fields, such as agriculture [15] or engineering, to develop new processes and new tools to manipulate matter at the atomic level [16]. For example, NMs can be used for the safe storage of hydrogen for use as a clean fuel [17]. In nanomedicine, NMs are applied in the mapping and diagnosis of diseases with the so-called “lab-on-a-chip” [18], the arrays of nanosensors [19], magnetic nanoparticles [20], and “quantum dots” [21,22]. Moreover, NMs can be applied as molecular nanostructures to reinforce asphalt and concrete [23,24]. Environmentally, NMs are also applied for purification and desalination [25], as well as in water treatment and control, in treating the effects of air pollution and in reducing the time of degradation of plastic [26]. In addition, NMs can be applied in food processing and respective storage, and as nutraceutical matrices [27,28,29,30,31]. 

## 2. Formulating Nanomaterials in Innovative Products

The increased use of NMs in the global industry has led to concerns about potential toxicological effects on the human health and the environment. Nowadays, the control and regulation of NMs is mandatory. Thus, the European Research Council and EURO-NanoTox have constituted a so called “nano-security group”, which has been appointed to assess the safety of a newly designed nanomaterial [11].

The European regulation for pharmaceutic and cosmetic products demands the confirmation of the nanosafety by special nanotoxicological tests [1]. The same holds for the agricultural and food sectors. It should be noted that the use of NMs as an ingredient in disinfectants, preservatives, pest control products, and others is subject to authorization based on a separate assessment of the nanospecific risks. Additionally, if there is no specific regulation about the use of NMs in chemical products, the product is subject to EC Regulation No. 1907/2006 (REACH) [11]. 

The authorities aim to emphasize reproducible studies in the context of a correct assessment of nano-security, meaning the need to establish a reproducible process to produce and test the biosafety levels of NMs [32]. The studies need to be well structured and optimized, with a precisely defined protocol for the possible understanding of the level of exposure and its distribution in the ecosystem and in the human body, thereby allowing the correct evaluation of the safety of the product [3,32]. Therefore, it will be essential to control and verify a series of characteristics and challenges before placing products on the market [1], including among others (1) the plasmonic properties, (2) gap band factor, (3) surface coating, (4) particle size, (5) surface electrical charge, (6) morphology, and (7) phase stability. 

The plasmonic properties of optically active nanoparticles or noble metals (e.g., gold, copper, and silver) possess strong interactions with the incident light and can amplify the local electromagnetic field, thus influencing some of the biological characteristics of the particles [33]. These optical NMs can convert light into thermal heat and, consequently, they can be used in photothermal therapy. It is worth underlining that during the periodic characterization of the interaction of the biological system, it is possible to generate false results, either positive or negative, influencing nanotoxicity [34]. This phenomenon should be the focus of further investigation.

The excitation of NMs with an appropriate band interval (gap band factor) can impact their optical and redox properties, as well as the risk of generating reactive oxygen species [35]. Moreover, the enthalpy becomes less negative, and the toxicity of the material increases as the energy of the conducting band of the metal oxide nanoparticles hydrates [36].

The surface coating affects the surface load, where it can either keep the particle stable or be designed such that it can recognize and react with specific bonds [37]. It can alter the reactivity of the particle and it governs many alternative functions, thereby stressing its importance for nanotoxicity.

The particle size governs the surface area and mass of the particle unit. Thus, the size of the nanoparticles is an important parameter and the most often studied. Density and surface area vary per unit of mass, which increases with size [37,38].

The surface electrical charge of the nanoparticles (positively or negatively charged) influences the interaction of the NMs with subsystems or biological membranes in an aqueous environment [32]. Logically, if the particle is negatively charged, it will be attracted to positively charged surfaces (and vice versa). Generally, the positively charged nanoparticles are recognized as more toxic and may even damage the membrane of cells. Moreover, they can interact with enzymes, proteins, and DNA [39] with an increased risk of genotoxicity [40,41].

The wide variety of shapes and morphologies of the NMs can affect their stability, transport, surface adsorption, and absorption by biological systems [42].

The large-scale phase diagrams do not predict the phases of the nanoparticles, as there is a difference in phase stability between the large-scale dynamics and the nanoscale [43]. This process is more frequent during the nanoparticle synthesis at the temperature below the melting point of the ingredients. The high curvature and the high surface area of NMs are the generators of this effect [44]. The synthesis methods used for the production of NMs may lead to a series of invalid features in the crystals and their densities [44]. The presence of impurities in NMs may increase their instability, leading to i.e., Ostwald ripening—a phenomenon that describes the change of an inhomogeneous structure over time [45]. This can be justified by the indirect proportionality that exists between the internal pressure and the diameter of the particles.

**Figure 3:**
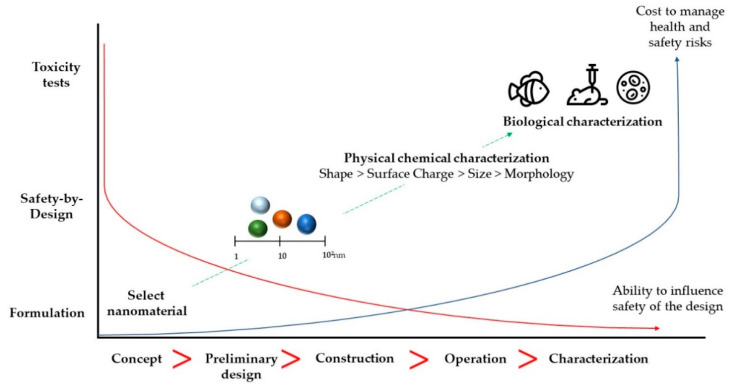
Relevant properties of nanomaterials in view of their safety assessment and the influence over a product’s lifecycle.

The control of the mean particle size, surface charge and chemical modification, morphology, porosity, and phase stability is instrumental for quality assessment and safety. The first techniques used for the physicochemical characterization of NMs are the Dynamic Light Scattering to measure the mean particle size and polydispersity index, and the zeta potential to provide information about the electrokinetic potential in colloidal dispersions estimating the stability of NMs over time and in different types of fluids. Microscopic techniques that characterize morphology include the Scanning Electron Microscopy and Transmission Electron Microscopy, depending on the size and material used in the production of NMs. Other techniques, as X-ray diffraction and thermal analysis (e.g., differential scanning calorimetry) are employed to observe specific characteristics as polymorphisms and chemical interactions. After physicochemical characterization, in vitro in selected cell lines and pre-clinical studies are usually followed (Figure 3).

## 3. “Safety-by-Design” of Nanomaterials

Nanomaterials (NMs) have different properties that may influence their toxicological profile [3]. Thus, official nanotoxicity guidelines are established to allow the safe design and application of NMs [32,41,46]. These include (1) the particle size, as the toxicity for and uptake by cells will increase with smaller sizes of the NMs; (2) the particle charge, as NMs with positive charge have a higher toxicity due to increased interactions with negatively charged biological surfaces [47]; (3) the ionic dissolution, as a higher ionic dissolution leads to a higher toxicity, and (4) the shape, as anisotropic or rod-shaped NMs are less efficient and can cause significant damage in the near infrared, which can destroy the target cells [32,48].

The main concern for nanotoxicologists is the protection of the human health while identifying the risk factors caused by NMs. This identification is a huge challenge, since people can easily be exposed to NMs that are integrated in compounds manufactured by uncontrolled, non-purified, and complex processes [49]. Therefore, care must be taken when assessing the potential risk of the projected nanomaterials [50], and the following outlines should be taken into consideration: (1) to select the NMs for nanotoxicology research and to create environments with exposure conditions (physical, chemical, and biotic) and exposure times; (2) to describe all of the characteristics of the means of exposure, as well as present metadata that allow cross-comparisons in detail; (3) to select forms of designed NMs (characterized by the process of a risk assessment in order to ensure a homogeneous assessment) with concentrations in scale; (4) to examine and choose several end points for the characterization of designed NMs (e.g., physicochemical properties and characterization of toxicity); (5) to quantify the load and compartmentalization of designed NMs, thereby enabling a comparison of the effective doses in biological response; (6) to follow experimental control treatments; and (7) to approach in the appropriate form of rapid screening in order to obtain relevant knowledge. It should be noted that most of the studies are carried out under specific conditions of exposure and they do not present highly significant results. Apart from the characterization and identification, several other risk factors of NMs include: (1) finding common processes that release large amounts of designed NMs (either due to the lack of large-scale nano-industry or due to the integration of NMs with other compounds); (2) the aggregation of NPs with other (non-toxic) compounds during the release process; (3) the risk identification of NMs during in vitro testing; (4) understanding the complexity of the interaction of NMs with biological systems; (5) the fact that the smallest difference between NMs can lead to major changes in the NMs interactions with biological systems in vivo; (6) the complexity of biological systems, associated with the great variability of NMs, as well as their dynamic transformation; (7) the lack of instruments, concordant protocols, and theoretical understanding that hinder the reproducibility of experiments; and (8) the lack of standardized experiments with biomarkers, demonstrative functional analyses, dosing, and statistics [51].

**Figure 4:**
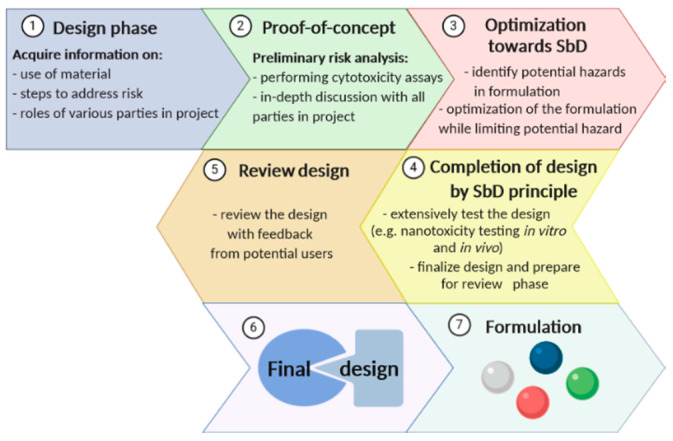
Schematic of the Safety-by-Design principle.

“Safety-by-Design” (SbD) is taken as a starting point when engineering a novel NM, thereby strictly following the principles of Drug Discovery and Development (DDD) throughout the whole process of product development [52]. The belief behind “Safety-by-Design” (SbD) is to engineer unwanted effects out of the NMs by implementing the knowledge of the NMs’ adverse effects on the environment and human health into the process of designing the desired NMs (Figure 4). In the construction industry, the main focus is on modifying the initial design in order to prevent accidents in the workplace and minimize the negative effects on the health. The development of processes that reduce the risk of the product toxicity have been observed as well in the field of green chemistry [53].

The NANoREG and NANoREG II, started in 2015, consist of two European projects that articulate the meaning and practice of SbD [53,54]. Firstly, functionality and safety are assessed in an integrated manner in the course of product development. Secondly, these rules are based on three pillars: safe design, safe production, and safe use. The support of the “safe innovation” approach, whereby the concept of SbD is combined with “normative readiness”, is a necessary information and knowledge within the innovation chain, ensuring compliance with the standards [52].

Security is the central concept of SbD, but it is important to note that absolute security is never reached. This may lead to the question of what is sufficiently safe, who should define the levels of the security, and how this decision is made. It follows that it may be more appropriate to use the expression “safer” instead of the absolute terms “safe” and “security”, for instance [53]. However, most of the projects continue to use the absolute terms of security and protection, thereby failing to recognize the relational value of this concept or the underlying challenges [53]. SbD tries to rephrase the question of safety to: “Is it inherently safe?” and “Can we design the product while ensuring its safety?” In this way, the political challenges regarding with standardized risk assessment are accepted. These aspects require a great interaction of technical, social, cultural, political, educational, and economic considerations. According to SbD, the collaboration between scientists and innovators can generate new challenges and concerns, although on the other hand, the result from these collaborations may allow the acceptance and realization of SbD, as well as affecting public confidence, both at the level of research and the level of products [53].

At all stages of the DDD process, the pharmaceutical industry uses several methods to assess the risks of toxicity. According to the REACH regulation, the focus on the toxicity tests occurs essentially as soon as the product has entered the market. However, it has been verified that the vision of SbD for the projected NMs has been approximated to the principles of DDD, instead of the current models of normative toxicology [55].

In DDD, in vitro and in silico screenings are carried out in order to avoid the toxicity and failures associated with safety in development. “Fail early, fail frequently” is the DDD discovery paradigm [56]. It motivates the attempt to reduce future losses without canceling the need to carry out toxicological assessments in vivo. In turn, early in vitro screening in DDD is performed with the objective of determining targets and non-targets in which a compound interacts in order to avoid side effects [57]. For this reason, it can also be called a pharmacological profile. However, despite the attempts to solve the toxicity problem, the safety concept designed for the drug is not complete. It is incorrect to state that all of the adverse events of a drug are known when it reaches the market. Until the drug is commercialized, it is carefully investigated, but there is no guarantee that they will not be removed later for safety reasons. For example, in the WITHDRAWN database, there are data that contain more than 500 drugs withdrawn from the market [58].

The implementation of SbD has pushed nano-security research away [53]. However, when SbD is presented as a way for nano-security to keep pace with innovation, it is assumed that it will require new toxicological tests and will reduce the time and workload required for “regulatory readiness”. This will depend on the predictability of the simplified tests. SbD tries to guarantee safety, despite the problems that are constantly arising. However, safety tests precede the identification of risks, which themselves precede the understanding of the mechanism; this in turn precedes the prognosis of SbD. Thereupon, “Safety-by-Design” cannot be used to overcome the problems that may arise after toxicological risk assessments for proposed nanomaterials.

## 4. Nanotoxicology: From Past Lights and Shadows to Current Concerns

The term nanotoxicology has only gained interest from the last two decades onwards [32,41,46]. Since that time, many advances have been made in this area. Two important factors led to a rapid progress in this branch of science [59]. Firstly, *“the large-scale production of diversified nanomaterials and remarkable progress in the development of new types of nanomaterials with disconcerting physical and chemical characteristics”* [60]. Second, many studies based on constantly improving NMs have stimulated research in Physics, Chemistry, and Bioengineering, leading to new interdisciplinary progress in Nanoscience and its applications. For example, there has been huge progress in the bioapplication of NMs [60]. 

Nanomedicine and nanotoxicology are strictly linked, since both can explore the same mechanisms and affect identical metabolic pathways [61]. Bearing in mind that newly NMs can exhibit specific toxicity, it is necessary to summarize and reassess the data accumulated from time to time, thereby ensuring safety [62]. The development of current nanotoxicology studies is surprising, mainly in the biological area [41,47,63]. For example, the biosynthesis of insecticidal nanoparticles mediated by plants and other botanical products is constantly developed [64,65,66].

Nanotoxicology has become a subdiscipline at the interface of toxicology and NMs [60]. Due to their extremely small size and large surface area to volume ratio, NMs have different properties compared to their larger equivalents that may enable unpredictable interactions with cells and tissues. Nanotoxicology tends to highlight the possible toxic interactions between NMs and different biological systems (cells, tissues, and living organisms) [32]. Several years of research have showed that the interactions of NMs with the environment and with cells of living organisms are highly complex [41,46,67,68]. However, it has not been revealed how the properties (both physicochemical and morphological) of NMs can influence these interactions [69,70,71]. 

The morphological and physicochemical properties of NMs have a great impact on the interaction with biological cells and may influence their toxicity. Nanotoxicology is responsible for the analysis of the toxic effects of NMs, especially since the materials’ size plays a significant role in the toxicity of NMs [10,72]. The concept of nanotoxicology is based on different parameters, such as the size, surface area, morphology, composition, surface chemistry, agglomeration/aggregation phenomena, etc. In fact, all of these parameters have a critical impact on the determination of the nanoparticles’ dose and consequently, the precise assessment of their toxicity. However, determining the maximum exposure values of toxic NMs would be impossible without in vitro and in vivo tests. Many strategies to study the nanotoxicology and the interaction of NMs with biological systems are already in place [41,46]. Initial studies on the toxicity of NMs were carried out in the last decade of the 20th century, already revealing that materials of a micrometric scale did not present toxicity, while materials at a nanometric scale might have some toxic effect [41,46,61]. 

## 5. Toxicity Tests

Toxicity tests can be performed on cell cultures (in vitro) and in living organisms (in vivo) such as fish, mice, or rats [73,74]. There are several standardized toxicological tests that are available to assess the biological response of a chemical substance. However, there is no standardization for the assessment of nanoparticles toxicity. It causes many difficulties in the comparison of the results regarding the toxicity of the tested ingredients. Most of the toxicity tests for NMs have been performed in vitro, using cultures of mammalian cells that were extracted from the most different parts of the body (e.g.,: brain, lungs, heart, skin and liver) [75]. Although in vitro tests are less expensive than in vivo and the results may be obtained in a shorter time, it is not possible to infer potential implications related with the human health based on the in vitro only [76].

Since continental and marine waters would be the main receiving compartment, in vivo tests were mainly carried out in aquatic organisms that would reflect the impact of nanomaterials on the environment [73]. During the contact with animals, the variation of the NMs concentration allows to calculate statistically the indicators that will allow the comparison of toxicity between different nanomaterials and/or between nanomaterials and traditional chemical substances. The most used evaluation parameters are [12,77] the LC_50_ (i.e., concentration of nanomaterial that causes the death of 50% of the population, LOEC (i.e., low concentration that causes a noticeable effect on the organism), and NOEC (i.e., maximum concentration, at which no effect is observed on the organisms). Moreover, experimental animal trials have advantages, with one of the important ones being the assessment of the kinetics of nanoparticles through absorption, distribution, metabolism, and excretion (ADME).

### 5.1. In Vitro

#### 5.1.1. 2D Models

The most commonly used method to determine NM toxicity on a 2D platform is the exposure of an NM dispersion to adherent cells, where the NMs are dispersed in the media. This method in itself is straightforward; however, obtaining a reproducible and homogeneous NM dispersion in the media poses a challenge. For example, many dispersions are diluted by sonication. Yet, the duration and strength of sonication are two parameters that influence the NM size distribution and agglomeration state and thus also influence the cellular uptake and NM toxicity greatly [78]. 

Another frequently used method is the NM surface presentation. In this method, cells are seeded on top of a NM layer, which is immobilized on a substrate. Thus, the NMs cannot agglomerate and remain static on the surface. Moreover, it offers additional control over the NM density on the surface and the NM dose per cell, thereby optimizing the cell to cell dose variability. 

One of the major drawbacks in the previous systems is the inability of using buoyant NMs. Inverted cell culture systems are preferable for these, since the cells are attached to a coverslip and suspended in the media from above [79,80]. Some NMs have shown nanotoxicity in this method and not in upright exposure where cells are attached to the bottom [80,81]. However, this method cannot be used for all NMs, since larger-sized or insoluble NMs will sediment over time and thus show limited nanotoxicity in inverted cell cultures [79,80,82].

The cell culture using an air–liquid interface is commonly used to assess toxicity for respiratory cells in vitro, and it is also regarded as a more cost-effective method compared to their in vivo counterparts [83,84]. In this method, the media is supplied to the cells from below. As such, the cell surface is exposed, which enables direct contact with the airborne NM, thereby more closely mimicking the in vivo lung situation [83,84,85]. 

#### 5.1.2. 3D Models

A co-culture comprises of two or more cell types (e.g., lung tissue composed of alveolar cells and macrophages). They are not necessarily 3D; however, they do provide more relevant information than a multitude of 2D models, since they offer additional cell–cell contact that promotes in vivo-like cell–cell interactions and cross-talk. These interactions and cellular responses provide a more relevant and in-depth NM toxicity research model. For example, the inclusion of macrophages into a tissue model not only enables research on the direct NM toxicity, but also the indirect toxicity mechanism where NM internalization by macrophages leads to an inflammatory response and subsequent tissue damage [86].

Spheroid microtissues consist of closely connected cells cultured together, thereby enabling the cells to aggregate into a tight three-dimensional ball. A spheroid introduces a more realistic, yet simplified, 3D architecture with more complex cell–cell interactions and an oxygen and nutrient gradient within the tissue. Moreover, the 3D spheroid in itself offers a barrier to NM distribution and cytotoxicity [86,87]. For example, it was shown that penetration of monodispersed drug–silica nanoconjugates was dependent upon size, with smaller NMs penetrating deeper in vivo [88]. A multicellular spheroid, or an organoid, resembles even more closely the in vivo organ [89,90]. These tissues have enhanced morphologies and show increased functional activity (e.g., cell communication or a greater secretion of proteins and molecules), which all play a role in the NM uptake and subsequent cellular response [86]. The InSphero^TM^ model comprising of primary human liver spheroids has even been used to study the nanotoxicity of a range of industrially relevant NMs (e.g., zinc oxide, silver, and TiO_2_) [89].

The incorporation of tissues or organs on a microfluidic platform (Organ-on-Chip; OoC) enables the evaluation of NM nanotoxicity in highly dynamic conditions in vitro [91]. An OoC provides more control and is able to more accurately mimic the in vivo microenvironment of the tissue. Moreover, it also provides more physiological control over the NM presentation and dosimetry. Since the NMs are dispersed in media, it also includes physical and chemical interactions that can occur between the NM and the media [92]. However, the small sizes of the platform lead to more dominant surface effects inside the channels, which can lead to surface adsorption of the NMs. Moreover, the preferred laminar flow inside the chip prohibits the solutions from mixing, which can be problematic when evaluating nanotoxicity. 

Precision-cut tissue slices (PCS) still retain the tissues’ native architecture and is thus a relatively novel approach to mimic the in vivo microenvironment [93]. It is compatible with a range of tissue samples from different species such as rodents and humans. PCS is especially popular for determining the nanotoxicity in lung [94] and liver tissue [95]. One study determined the nanotoxicity from a range of industrial relevant NMs (e.g., TiO_2_, ZnO, Ag, and multi-walled carbon nanotubes) [96]. Despite the wide range of use, slicing of the tissue does result in tissue damage on the surface which can generate inflammatory responses. This can interfere with the results of subsequent studies. Moreover, only a limited number of slices can be generated from one organ [97]. Advantages and drawbacks of various in vitro methods are summarized in Table 1.

### 5.2. In Vivo

#### 5.2.1. Aquatic Models

A popular biomethod to screen for NMs toxicity internationally is the toxicity test with freshwater *Daphnia magna* and *Dapnia pulex,* which are small planktonic crustaceans. These methods have aided in determining the exact mechanism of acute and chronic nanotoxicity. Moreover, due to the strict guidelines and standards of the test, the NM effect on mortality and reproduction is easily monitored. It should be noted that crustaceans are primarily used as a pre-screening method, as the biological differences between humans and these crustaceans are too large to conclude anything final on nanotoxicity [98]. 

The most widely used aquatic biological and nanotoxicity model are zebrafish, which have a similar developmental process and genome to mammals and also humans [100]. Since embryogenesis occurs outside of the womb and the embryos are transparent, the developmental period is easily monitored. Moreover, embryogenesis is rapid, with structures as the brain, muscle and skeleton already formed 24 h post fertilization (hpf), and sensory organs such as eyes and ears formed after 5 days post fertilization. The genome of the zebrafish has also been mapped completely, which allowed the production of standardized microarrays for nanotoxicological studies. This enables researchers to perform rapid and large-scale nanotoxicity experiments. Moreover, the use of zebrafish allows testing of NMs through multiple exposure routes, including direct dispersion in water. However, the rapid developmental process also makes it difficult to perform systemic embryo-based nanotoxicity assays [101,102,103,104].

#### 5.2.2. Small Rodents

The most frequently used in vivo models to determine nanotoxicity are still mammal models, especially small rodents such as mice, rats, and rabbits. These are mainly used because of their close resemblance to humans, they are easier (and cheaper) to be maintained than larger animals such as pigs, which are genetically very close to humans [105]. Many different toxicity studies have been devised, including single and multiple administration to assess acute toxicity and chronic nanotoxicity, respectively. The Organisation for Economic Co-operation and Development (OECD) has even devised guidelines to evaluate the in vivo oral, dermal, and LD_50_ nanotoxicity. Moreover, small rodents can be used to test many different exposure routes, including NM inhalation, intravenous or intraperitoneal injections, ingestion, intra-tracheal installation, or gavage. The most used techniques for exposure to receptors are intravenous and the inhalation of nanoparticles [106]. The main concern regarding nanotechnology is the access of nanoparticles to the circulatory system. Therefore, some studies have already simulated this situation by injecting suspensions of nanoparticles directly into the bloodstream of rats [107]. In these tests, healthy animals with similar mass receive known doses of a given nanoparticle suspended in a certain non-harmful liquid. Usually after 48 h of the administration of the particles, blood samples are taken for cell counting (such as monocytes, granulocytes, lymphocytes, platelets, red blood cells). Additionally, the measurements of hemoglobin concentration are conducted. This type of test can also be used to assess the distribution and/or bioaccumulation of nanoparticles in vital organs. Moreover, thanks to this procedure, it is also possible to evaluate the distribution of the nanomaterial in the body, which is important for the pharmaceutical industry in the case of purpose of using the particles as nanotransporters [108]. Most of the studies that have searched for the correlation between the effects of nanoparticles inhalation were carried out in vitro using cells of bronchi and alveoli of lungs [109]. On the other hand, in vivo injections using suspensions of nanoparticles applied into the trachea of rats were also used. Thereupon, the relevant changes in the respiratory system might be observed [110]. The trend in in vivo tests has pointed to the simulation of contaminated atmospheres, as was observed by Bermudez et al., who have exposed mice, rats, and hamsters to a TiO_2_ aerosol inside a sealed container under normal oxygenation conditions, thereby observing inflammation of the alveoli [111]. Advantages and drawbacks of various in vivo methods are summarized in Table 2.

## 6. The Impact of Nanoparticles: In Vitro and In Vivo Studies

Titanium dioxide and silver nanoparticles are the most commonly used nanomaterials due to their versatility and functionality in various areas as health, environment, and industries with a positive impact in the economy.

### 6.1. Titanium Dioxide Nanoparticles

Titanium dioxide (TiO_2_) is used in various materials (e.g., paper, plastic, ceramics, rubber, printer ink, floor coverings, catalysts, fabrics and textiles, cosmetics, food colors, medicines, paints, coatings, and electronic components). The different types of TiO_2_-based NMs can be distinguished by four different crystalline structures: rutile, anatase, brookite, or titanium dioxide [112], with rutile and anatase the most frequently used in products consumption [113]. 

The National Institute for Occupational Safety and Health (NIOSH) has recommended the general exposure limits of 0.3 mg/m^3^ of air for the nano form of TiO_2_ [114]. According to the review presented by NIOSH, five epidemiological studies have already been carried out on the TiO_2_ NPs exposure of workers, which showed no relation between TiO_2_NPs and the mortality or morbidity from lung cancer. Thus, NIOSH have considered that there is insufficient evidence to classify TiO_2_NPs as potential carcinogens in an occupational context. However, according to the classification of the International Agency for Research on Cancer (IARC), TiO_2_NPs are considered as “possibly carcinogenic to humans”; since TiO_2_NPs have shown carcinogenic effects and genotoxicity in animals, its genotoxic potential has also confirmed [114,115]. Moreover, present in vitro tests using bacteria have not proved genotoxicity induced by titanium dioxide, while the positive effects have been reported in eukaryotic cells and in animals [116].

In addition, TiO_2_ has attracted more and more attention as an effective nanomaterial for the photocatalytic reduction of carbon dioxide (CO_2_) [117]. It has been combined with intermittent and renewable solar energy storage in the form of connections chemicals in order to convert anthropogenic CO_2_ gas into fuels. Recently, there has been considerable progress in the improvement of the performance of TiO_2_ photocatalysts in reducing CO_2_ [117].

In 1997, the accumulation of TiO_2_NPs and the alteration of gene expression were studied by a zebrafish model (Danio rerio). The results revealed that long-term exposure to low concentrations of TiO_2_NPs is toxic to the reproductive system of this species [73]. Moreover, they concluded that long-term exposure of these nanoparticles is similar to that caused by many toxic reproductive substances, such as endocrine chemicals. In 2010, scientists demonstrated the transfer of TiO_2_NPs from the water flea (Daphnia) to the zebrafish through the freshwater food chain [118]. These researchers have also found that prolonged exposure to low doses of TiO_2_NPs can alter the reproduction of certain aquatic organisms. Ultimately, they showed that these reproductive changes can cause disturbances in the population dynamics of these organisms in aquatic environments. It was also observed that prolonged exposure to TiO_2_NPs negatively affected the reproduction of zebrafish [119], as well as the survival of embryos, regardless of their concentration [102,120]. Moreover, histological analysis and a study on the gene expression showed a delay in folliculogenesis (maturation of the ovarian follicle), as well as changes in the maturation and function of the ovaries of fish exposed to TiO_2_NPs. Thus, it has been confirmed once more that these nanoparticles can be toxic at the level of the reproductive system of this species [119].

These results suggest that the contamination of aquatic environments with TiO_2_NPs, even at low doses, may have an impact on the reproduction of species [121]. The effect should constitute another reason for investigation the potential impact of TiO_2_NPs on the environment and the human health [119].

### 6.2. Silver Nanoparticles 

Silver nanoparticles (AgNPs) are currently one of the most studied nanomaterials [20,64,65,122,123,124]. These nanoparticles are the most used from an industrial and commercial point of view, due to their characteristic properties (e.g., chemical stability, malleability, flexibility, high electrical and thermal conductivity, catalytic activity, relatively low production cost, and antimicrobial action against bacteria, viruses, fungi, and protozoa). Thus, AgNPs are widely used in various applications and commercial products, such as antibacterial, antifungal, antiviral, anti-inflammatory, anti-tumor, regenerative, biosensor, and catalyst agents [125]. Moreover, they are also present in products in the food, textile, perfumery, pharmaceutical, agricultural, hygiene, cleaning products, paints, electronics industries, among others, leading to human and environmental exposure [126]. Furthermore, AgNPs have wide applicability in medicine, e.g., to cover the medical devices, to prepare the nanogels, or to produce the formulations for skin lesions [64,65,123,127].

The control of the parameters during the synthesis of AgNPs is necessary to guarantee the reproducibility of their production. The toxicity of AgNPs depends on several parameters, such as the preparation of the colloidal suspension, the aggregation state, the chemical nature of nanoparticles, the dose, the nature of the living organism, the cell type, the morphology, and the surface size [128,129]. 

In general, it has been noticed that low concentrations of AgNPs show insignificant toxicity within in vitro and in vivo assays. However, with the increase of the AgNPs concentration, the toxicity of nanoparticles and their accumulation in various tissues/organs tend to increase. In high concentrations, the inhalation or ingestion of AgNPs can cause adverse effects and even lead to tissue death [128].

Currently, toxicological studies of AgNPs in zebrafish are still in the exploration phase. Griffittet al. (2008) [130] have measured the toxicity of metallic and soluble nanoparticles in zebrafish using static 48-hour bioassays. The obtained results have indicated that the 48-hour LC_50_ of AgNPs (26.6 nm) was 7.07 and 7.20 mg/L for adult and juvenile zebrafish, respectively. The LC_50_ of 48 h of AgNPs (18 nm) in deionized water in embryos after three days of incubation with zebrafish was 0.94 mg/L. Moreover, scientists have investigated the acute toxicity of poly vinyl pyrrolidone (PVP)-coated AgNPs (81 nm) to zebrafish and found that the LC50 value of 48 h was 84 μg/L. The different LC_50_ values of 48 h of AgNPs in these studies stem from the different parameters on which the toxicity of AgNPs is dependent. The stability of citrate-capped AgNPs and their toxicity on embryonic development of zebrafish have been assessed, showing that large aggregates of AgNPs were relatively non-toxic compared to nanosized particles. Thus, toxicity to zebrafish is based on the size of the particle.

In the oral toxicity study, AgNPs with the size of 56 nm were analyzed for 90 days of exposure (doses varying from 0 to 500 mg/kg). Significant decreases in the body weight of male rats were noticed, while there were no changes in food or water consumption during the exposure period. The scientists concluded that the liver was a target organ of AgNPs, both in male and female rats. Adverse effects were observed with doses of 125 mg/kg and above [131]. Pinzaru et al. evaluated the toxicity of poly ethylene glycol (PEG)-coated AgNPs in vitro against human keratinocyte cell culture (HaCat) and in vivo in non-invasive tests on mice (rat species) without SKH-1 [132]. The in vitro results showed that at concentrations between 0.1 and 3.0 µmol/L, the number of viable cells was not affected during different periods (24, 48, and 72 h). In turn, the concentrations of 10 and 50 µmol/L have showed a cytotoxic effect induced by nanoparticles, where in 48 and 72 h, the effect was more significant. In turn, the in vivo tests using mice, where a dose of 10 mg/kg of AgNPs was administered daily for 6 days, showed that the intraperitoneal injection route did not induce harmful symptoms. 

## 7. The Importance of Nanosafety

Nanosafety refers to the assessment of risks to the human health and the environment, as well as to the ecological risks arising from the use of designed nanomaterials and the evaluation of their toxicity level [133,134]. The issues facing nano-security stem from the fact that there is often a barrier between the two scientific communities, namely in the field of ecology and ecotoxicology. It is possibly related with customs and methodologies; however, in the assessment of the human toxicity, it is important to communicate between researchers in order to reduce the duplication of efforts and to optimize resources, processes, or research methods [134].

In the last 20 years, studies regarding the nano-security have considerably increased. Some of the studies are contradictory, and they also do not clarify the safety of nanomaterials [135]. Even though it was possible to determine that several nanomaterials were able to penetrate the lungs and gastrointestinal tract, only a small part actually reached the bloodstream and was distributed throughout the body to secondary target organs. The vast majority of nanomaterials are captured by macrophages in the lungs or excreted through the feces [51].

According to nanotoxicologists, certain criteria have demonstrated suitable outcomes regarding the characterization and assessment of toxicological risk for carbon nanotubes and fullerenes [136,137,138]. These criteria stemmed from the most common mistakes committed by the researchers: (1) insufficient characterization often traced back to the supplier, e.g., if the particle size declared by the supplier is incorrect, it will lead to significant errors in subsequent experiments; (2) inadequate testing for contamination of the analyzed nanomaterial. It is important to note that in most of cases, the samples were not treated in sterile conditions; e.g. during the tests that analyze the inflammatory processes, the samples could be contaminated with endotoxins, which initiated the same reaction as inflammatory mediators. Consequently, false positive results are obtained; (3) the interactions of solvents and dispersing agents with the test system are not taken into account; and finally, (4) the lack of control tests, which leads to incomplete conclusions.

The vast majority of the publications have keywords associated with “the toxicological effect”, even when the study did not actually prove it due to experimental errors [139]. The lack of conclusive and confirmed data reduces the quality of determining the safety of nanomaterials For example, conducting animal tests to determine the appropriate dosage and to characterize the toxicological risk of NMs for the environment and for the human health is insufficient [140]. Moreover, it has been inferred that laboratories should undergo a standardization process and quality control to reduce any duplication of work. Unfortunately, this phenomenon has become difficult to avoid. With the standardization of epidemiological studies, laboratories should select a sample for using a universal model to allow a comparison of the parameters under evaluation. The analysis of the obtained results must be in accordance with the principles of Good Laboratory Practices and should be carried out by qualified scientists. It seems that these procedures are not taken seriously, since in most cases, the Standard Operating Procedure is not followed, thereby significantly endangering the safety of NMs.

Nanotoxicologists have assessed that not all nanomaterials are created the same way, which means that even when the differences in the properties of the materials are insignificant, differences in the biological response can still occur [133]. The rapid growth in the number of nanomaterials have raised concerns about possible toxic effects on human health and the environment [141]. The greatest concerns stem from the application that requires direct contact with biological systems and those that are part of the constituents of medical devices, as well as pharmaceutical and cosmetic products.

Despite of all the knowledge on nanotoxicity accumulated in the last decade, it is still difficult to predict the bioavailability, biodistribution, degradation, elimination, and biological activity of nanostructures beforehand [142]. However, safety procedures in the manufacture, commercial, and medical industry have been established. Hence, new NMs would benefit from a comprehensive understanding of their toxicological mechanisms and their interaction with the biological system. These studies can illuminate the relation between nanoparticles and its impact on cells (e.g., nanoparticle–cell interactions, endocytosis, and intracellular traffic) [143,144]. There are some biological models that are available to check the toxicity of substances or agents in vitro and in vivo. In vitro models are mainly based on isolated cells and can be used in test tubes exposed to the tested substance. In turn, in vivo models are applied to the life organisms in order to observe changes in their development (growth, reproduction, mortality, etc.), which are toxicity indicators. The integration of metabolomics and transcriptomics in nanotoxicity studies is emerging [145].

The tests for NMs need to be reassessed with the perspective of building an appropriate method for the determination of nanotoxicity. In this way, the approaches of biological systems are considered and progressively applied in nano-ecotoxicological sciences. 

## 8. Conclusions 

Nanotechnology-based research is intimately related to nanotoxicology and nanosafety. Both of these latter disciplines are complementary and are mainly concerned with the study of the toxicity of nanomaterials and aim to improve the quality of the human life. A constant development in areas such as medicine can bring many benefits for human life and health. However, nanomaterials are being used by several industries and are thus present in many products. Nanomaterials may cause an array of potential toxicological and hazardous effects. Moreover, they can have detrimental effects on many ecosystems. The toxicological risks of using nanomaterials need to be screened, to map the potential risk of causing undesirable effects on the human health and on the environment. Based on recent scientific reports, it has been observed that commonly used methods have helped to design more effective nanomaterials while keeping the hazards of the substances at a minimum. In nanomedicine, the significance of nanotoxicology is particularly important to avoid the toxicity of drug nanocarriers.

## Figures and Tables

**Table 1:** The advantages and drawbacks of the various in vitro methods.

Type	Advantages	Drawbacks	References
2D cell culture			
Upright NM exposure	Easy experiment set-up; Can be used for virtually all 2D cell cultures;	Agglomeration of nanoparticles; Inconsistent protocols between studies; Inhomogeneous distribution over time	[78]
Surface-based NM presentation	Exact NM/µm; No agglomeration of particles; Homogeneous distribution over time Easy monitoring of uptake and toxicity	NM–substrate interactions influence internalization and toxicity results; Only static conditions can be tested.	[99]
Inverted cell culture	Assessment of buoyant NM nanotoxicity	Limited use for larger-sized or insoluble NMs	[79,80,81,82]
Air liquid interface	More physiologically relevant; Cheaper than in vivo studies; Range of commercially devices available	Limited to airborne NMs; Only relevant to nanotoxicity studies related to inhalation	[83,85]
3D cell culture			
Co-culture	Promotes in vivo-like cell–cell interactions; More relevant than 2D nanotoxicity platforms;	Still lacks 3D microenvironment	[86]
Spheroids and organoids	More in vivo-like complexity; Oxygen and nutrient gradient; Barrier to NMs distribution and nanotoxicity; Easy-to-use protocols	Heterogeneity; Lower reproducibility; Simplified 3D architecture; No high throughput	[86,87,89,90]
Organ-on-Chip	High throughput; Low cost; Physiologically relevant microenvironment; Precise control over NM presentation and dosimetry	Surface effects stemming from small dimensions; Little mixing of solutions; Difficult integration of sensors;	[91,92]
Precision-cut tissue slices	Compatible with a range of tissue samples and animal species; High reproducibility; Quickly obtainable; Retain the tissue native architecture	Tissue damage due to slicing; Limited number of slices per organ	[93,94,95,96,97]

**Table 2:** The advantages and drawbacks of the various in vivo methods. ADME: Absorption, distribution, metabolism, and excretion.

Type	Advantages	Drawbacks	Source(s)
Aquatic models			
Planktonic crustaceans	Standardized protocols and guidelines; Easy implementation	Primarily used as pre-screening method; Large biological difference to humans	[98]
Zebrafish	High throughput; Similar genome; Rapid developmental process; Low cost; Easy monitoring of embryogenesis; ADME effects studied	Difficult monitoring of rapid developmental process; Ethical concerns; Species to species variation	[100,101,104]
Mammal models			
Small rodents	Multiple routes of exposure; Guidelines exist to evaluate nanotoxicity; ADME effects studied; Chronic effects studied	Ethical concerns; Species to species variation; Expertise necessary; High cost	[105,106]
